# The vertebrate taxonomy ontology: a framework for reasoning across model organism and species phenotypes

**DOI:** 10.1186/2041-1480-4-34

**Published:** 2013-11-22

**Authors:** Peter E Midford, Thomas Alex Dececchi, James P Balhoff, Wasila M Dahdul, Nizar Ibrahim, Hilmar Lapp, John G Lundberg, Paula M Mabee, Paul C Sereno, Monte Westerfield, Todd J Vision, David C Blackburn

**Affiliations:** 1 Department of Ecology and Evolutionary Biology, University of Kansas, Lawrence, Kansas, USA; 2 National Evolutionary Synthesis Center, Durham, North Carolina, USA; 3 Department of Biology, University of South Dakota, Vermillion, South Dakota, USA; 4 Department of Biology, University of North Carolina, Chapel Hill, North Carolina, USA; 5 Department of Organismal Biology and Anatomy, University of Chicago, Chicago, Illinois, USA; 6 Academy of Natural Sciences, Philadelphia, Pennsylvania, USA; 7 Institute of Neuroscience, University of Oregon, Eugene, Oregon, USA; 8 Department of Vertebrate Zoology and Anthropology, California Academy of Sciences, San Francisco, California, USA

**Keywords:** Data integration, Evolutionary biology, Paleontology, Taxonomic rank

## Abstract

**Background:**

A hierarchical taxonomy of organisms is a prerequisite for semantic integration of biodiversity data. Ideally, there would be a single, expansive, authoritative taxonomy that includes extinct and extant taxa, information on synonyms and common names, and monophyletic supraspecific taxa that reflect our current understanding of phylogenetic relationships.

**Description:**

As a step towards development of such a resource, and to enable large-scale integration of phenotypic data across vertebrates, we created the Vertebrate Taxonomy Ontology (VTO), a semantically defined taxonomic resource derived from the integration of existing taxonomic compilations, and freely distributed under a Creative Commons Zero (CC0) public domain waiver. The VTO includes both extant and extinct vertebrates and currently contains 106,947 taxonomic terms, 22 taxonomic ranks, 104,736 synonyms, and 162,400 cross-references to other taxonomic resources. Key challenges in constructing the VTO included (1) extracting and merging names, synonyms, and identifiers from heterogeneous sources; (2) structuring hierarchies of terms based on evolutionary relationships and the principle of monophyly; and (3) automating this process as much as possible to accommodate updates in source taxonomies.

**Conclusions:**

The VTO is the primary source of taxonomic information used by the Phenoscape Knowledgebase (http://phenoscape.org/), which integrates genetic and evolutionary phenotype data across both model and non-model vertebrates. The VTO is useful for inferring phenotypic changes on the vertebrate tree of life, which enables queries for candidate genes for various episodes in vertebrate evolution.

## Background

Integration of data about organisms almost always requires a taxonomic framework. The Phenoscape project aims to integrate morphological and genetic data, incorporating information from both model organism databases and data from the literature on non-model organisms, including extinct taxa. Phenoscape requires a semantically defined taxonomic resource that includes extant and extinct species that recognizes both valid names and synonyms as they are used by different authors, and that is in line with current phylogenetic understanding.

Phenoscape’s initial focus is on data from vertebrates. Although vertebrates comprise only a small fraction of all biodiversity, the group is sufficiently large that the relevant taxonomic information is distributed among several different resources. We combined information from multiple sources to build the Vertebrate Taxonomy Ontology (VTO; http://purl.obolibrary.org/obo/vto.owl). As of October 2013, the VTO contained 106,947 terms annotated with 104,736 synonyms, 162,400 cross-references to other taxonomic resources, and 22 ranks.

Here we discuss the three main challenges encountered while building the VTO and our approaches to solving them: (1) extracting and merging names, synonyms, and identifiers from different and often conflicting sources; (2) structuring names in hierarchies based on phylogenetic relationships and the principle of monophyly; and (3) automating this process to accommodate updates in the source taxonomies and to maintain clear provenance for terms.

## Content and construction

### Selection of sources

The VTO does not seek to publish new taxonomic names, and thus we import names from existing source taxonomies. Sources have been selected based on their coverage, availability, and whether they are recognized by specialists as authorities for specific taxonomic groups. All the current sources are electronically available and either have compatible terms of use or have been made available for use and redistribution with permission.

**Figure 1:**
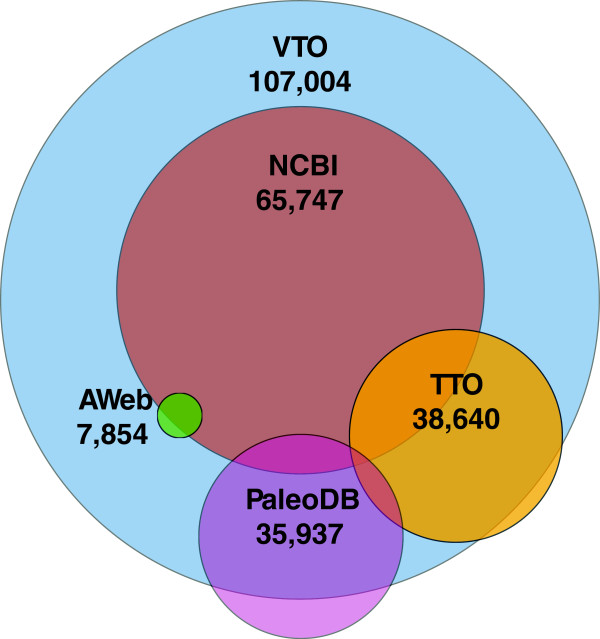
**Venn diagram showing overlap across source hierarchies used in the construction of the VTO.** Colored circles are scaled to the relative sizes of the source hierarchies and the VTO, with numbers indicating the number of terms in each hierarchy. Amount of overlap between colored circles indicates the relative amount of overlap in different hierarchies. VTO=Vertebrate Taxonomy Ontology, AWeb=AmphibiaWeb, NCBI=NCBI taxonomy, PaleoDB= Paleobiology Database (vertebrates only), TTO= Teleost Taxonomy Ontology.

VTO is currently built upon two resources with broad taxonomic coverage of the vertebrates and two resources with richer coverage of particular subgroups. The National Center for Biotechnology Information (NCBI) taxonomy provides the hierarchical backbone for extant taxa. Because NCBI taxonomy largely includes only species associated with archived genetic data, it excludes many extant and nearly all extinct taxa (Figure 1). To complement NCBI, we also incorporated taxonomic information across the vertebrates from the Paleobiology Database (PaleoDB). The Teleost Taxonomy Ontology (TTO) and the AmphibiaWeb (AWeb) taxonomy were incorporated to provide a more authoritative hierarchy and a richer set of names for specific taxonomic groups.

### National center for biotechnology information (NCBI) taxonomy

The NCBI taxonomy is a curated consensus view of taxonomic relationships [1]. It offers broad coverage with a coarse hierarchy and provides valuable linkages to molecular data. As of May 2013, the NCBI contained 65,747 taxonomic names for vertebrates.

### Teleost taxonomy ontology (TTO)

The TTO is a taxonomy ontology based on the Catalog of Fishes (CoF, [2]). It is subsequently modified by contributions from taxonomic experts as part of the Phenoscape project [3]; to date, 754 terms have been added (mostly genera, species, and some extinct taxa) that are not present in CoF. The TTO is updated periodically as needed to reflect both changes in CoF and to incorporate additional taxa encountered during the process of curation in Phenoscape [4]. These include taxa known only from fossils, additional names that we treat as synonyms of valid names such as misspellings or subjective synonyms (for example, names for which specimens are not available for making objective decisions about synonymy), and names used as placeholders in manuscripts or publications prior to a formal taxonomic treatment (e.g., “*Danio* aff*. dangila* (Fang 2003) [5]” or “*Agoniates* sp. (Toledo-Piza 2000) [6]”). Specimens that are not given a species designation are assigned to a nonspecific taxon in TTO that includes a citation to the curated publication (e.g., “*Eigenmannia* sp*.* (Fink and Fink 1981)”). This standard enables reasoning to be applied to specimens described in the literature that are excluded from traditional taxonomies such as CoF due to uncertainty in species affinities at the time of publication. A tool called ‘TTOUpdate’ (http://phenoscape.org/wiki/TTOUpdate_tool), developed by the Phenoscape project, can update TTO automatically with each new release of the CoF. As of May 2013, the TTO contained 38,640 taxonomic terms for valid species and higher taxa and 60,028 synonyms (taxonomic synonyms and vernacular names, the latter obtained from FishBase [7]). The VTO obtains taxonomic information from the TTO for taxa within the Actinopterygii, Chondrichthyes, and the agnathan clades Myxiniformes and Petromyzontiformes, and relies on NCBI for the hierarchy of the small number of remaining fish taxa (27 taxa, mostly Sarcopterygii).

### AmphibiaWeb (AWeb)

For living amphibians, we have chosen to graft the AmphibiaWeb taxonomy (obtained from http://amphibiaweb.org/amphib_names.txt) onto the NCBI backbone. The hierarchy and taxon sampling of the AmphibiaWeb taxonomy is more expansive than that of NCBI for this clade which contains fewer than 5,200 named taxa (as of October 2013). It is updated frequently, available online, and widely used. As of May 2013, AmphibiaWeb contained 7,854 taxonomic names, all of which are incorporated into VTO.

### Paleobiology database (PaleoDB)

The Paleobiology Database (PaleoDB, http://paleodb.org) is an expert-curated collection that attempts to cover the entirety of the fossil record, including information about taxonomy, specimen locations, and stratigraphic distributions [8]. Its primary use is as a repository of fossil occurrence data to support large-scale paleobiogeographic analyses. Thus, in addition to named extinct and some extant species, it includes data on trace fossils and body fossils that can be identified only at a higher level. The PaleoDB provides not only a listing of all currently published taxon names, but also other identifiers (such as long-obsolete synonyms and trace fossil taxa) that were not included in the VTO because they would not be encountered in the relevant literature. We also did not retain taxa from PaleoDB that are invalid, synonyms, or difficult to place because they lacked a parent classification. As of May 2013, we have incorporated 35,937 of the 39,180 vertebrate taxa (excluding trace fossils) of all ranks in PaleoDB, 28,451 of which are extinct.

### Constructing the VTO

**Figure 2:**
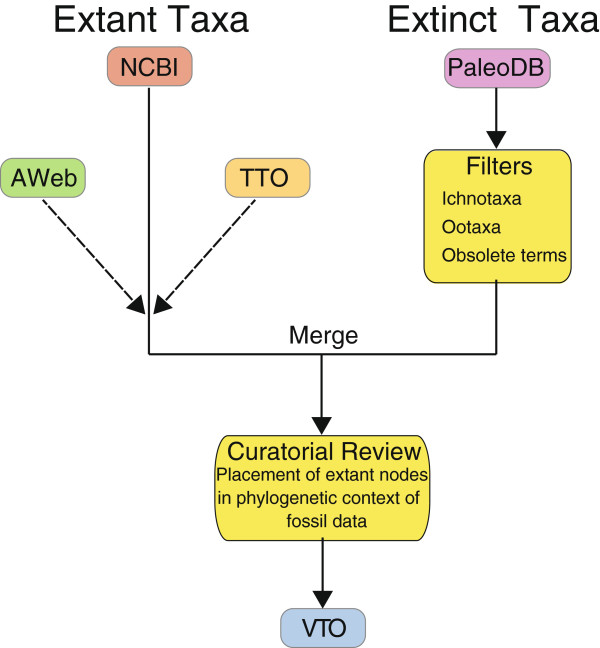
**Illustration of the construction of the VTO from source ontologies.** AWeb and TTO are grafted (dashed lines) onto the NCBI backbone; PaleoDB taxa are filtered prior to merging with NCBI. AWeb = AmphibiaWeb, NCBI = NCBI taxonomy, PaleoDB = Paleobiology Database (vertebrates only), TTO = Teleost Taxonomic Ontology.

We have followed the basic principle of phylogenetic taxonomy that supraspecific taxon names should, whenever possible, represent monophyletic groups. Because our knowledge of the Tree of Life remains imperfect and based on many independent phylogenetic analyses with both different data and different taxon sampling, curators must exercise expert judgment when making changes to reconcile conflicts between studies during the maintenance and updating of the VTO. The construction process of the VTO is initiated by importing the NCBI hierarchy. We developed a Taxonomy Ontology Tool (http://github.com/NESCent/Taxonomy-Ontology-Tool) to graft specialized taxonomies at nodes in a designated backbone hierarchy (here, NCBI’s) while merging lists of synonyms from multiple sources (based on matches between primary names). In this way, the Teleost Taxonomy Ontology (TTO) is used to replace the NCBI taxonomy under the nodes ‘Actinopterygii’, ‘Chondrichthyes’, the agnathan clades Myxiniformes and Petromyzontiformes, and AmphibiaWeb to replace the node ‘Amphibia’ and its descendants (Figure 2). This results in the portion of the VTO relevant to extant taxa and creates a framework (the “proto-VTO”) within which to add extinct taxa. Following the practice of both Catalog of Fishes and AmphibiaWeb, we treat subspecies as synonyms of their respective species.

The procedure for incorporating PaleoDB involves more expert interaction than either the TTO or the AmphibiaWeb taxonomy, and is thus not as amenable to automation. This is a result of three factors: (1) the PaleoDB taxonomic data must be grafted at many different nodes otherwise represented by extant species from other sources because extinct taxa occur across the vertebrate tree of life; (2) for some fossil taxa there is considerable taxonomic uncertainty; and (3) there are internal inconsistencies in the PaleoDB taxonomy and hierarchy (for example, the taxon *Rahonavis* and its former name *Rahona* both occur as valid taxa but in different parts of the taxonomic hierarchy). Thus, due to the large investment of time and effort required, we opted to integrate the PaleoDB taxonomy in a manner that was driven by our curation needs. PaleoDB subtrees are grafted at the lowest ranking node inclusive of that subtree within the proto-VTO. Taxa marked as “disused” in PaleoDB are then marked in VTO as obsolete, and any children of disused taxa from PaleoDB that are not themselves disused are attached at the root. We do not include names from PaleoDB that are associated with non-diagnostic material for biological species identification (e.g., track fossils, eggshells, or scales). Based on published taxonomic treatments, we have augmented parts of the hierarchy by moving or merging a small number (~50) of nodes. These changes, along with the rationale and the reference, were recorded in the “comment” annotation section for each VTO term modified in this way.

### Modeling of taxa and rank

We provide information about taxonomic rank for those VTO terms for which a rank has been provided in one of the source taxonomies. However, terms need not have a rank and we note that rank-free taxonomies are gaining traction in the literature. To annotate taxon terms with their taxonomic rank, we constructed a new vocabulary of taxonomic rank terms, the Taxon Rank vocabulary (http://purl.obolibrary.org/obo/taxrank.owl). The vocabulary consolidates the rank terms used by the NCBI taxonomy with those proposed in the Biodiversity Information Standards (TDWG) TaxonRank vocabulary (http://rs.tdwg.org/ontology/voc/TaxonRank). The resulting vocabulary contains 59 terms with links back to the corresponding terms in the source vocabularies. We maintain this vocabulary separately from VTO to promote reuse by other projects.

The VTO follows the same modeling pattern used to render the NCBI taxonomy in OBO format and in its OWL conversion (http://purl.obolibrary.org/obo/ncbitaxon.owl). In this pattern, each taxon is modeled as an ontology class, and ranks are assigned to taxon classes using the ‘has_rank’ annotation property declared within the Taxon Rank vocabulary.

### Synonyms

Taxonomic names can undergo multiple status revisions. As a consequence, taxonomic names encountered in legacy literature may not be in current use. Further, at any given time, multiple authors may use different scientific names to designate the same biological species; for example, two scientific names of the American Bullfrog are in common use today (*Rana catesbeiana* and *Lithobates catesbeianus*). Supporting integration of species-related annotations requires inclusion of all synonyms encountered in the literature irrespective of the official taxonomic status of that name (e.g., junior synonym, spelling variant). If a name is unavailable based on criteria in the International Code of Zoological Nomenclature [9], we do not incorporate it as a term into the VTO, but instead include such names as synonyms, whenever possible, to create more ways for users to locate information of interest.

Additionally, failure to find a match for a taxon term in the literature can be due to misspelling or use of common (vernacular) names. Common names are valuable for making the data organized by the VTO more readily accessible to non-expert users. Because each source taxonomy has its own mechanism for including common or vernacular names, these names have been merged into the VTO from each source. For instance, in the TTO, approximately 14,400 English common names were generously provided by Fishbase [7]. We take advantage of the author-defined ‘type’ tags for synonyms in OBO to define tags that distinguish the different kinds of synonyms such as ‘COMMONNAME’ and ‘MISSPELLING’. The VTO includes an additional annotation property ‘is_extinct’ to indicate taxa that are known only from fossil evidence or if such designation is present in a source taxonomy.

We have followed the principle that supraspecific taxon names should, whenever possible, represent monophyletic groups. We have made and recorded additional adjustments to the resulting hierarchy (especially coming from PaleoDB) to ensure that the VTO is consistent with our understanding of vertebrate phylogeny as established by current research.

## Discussion

Integrating ontology-linked biodiversity data to leverage the power of machine reasoners and other ontology-based computational tools requires taxonomies in the form of ontologies. In Phenoscape, such data integration allows us to link naturally occurring phenotypes among diverse taxa to phenotypes resulting from genetic manipulations in model organisms [3]. Curation of phenotypes in Phenoscape entails translating phenotype descriptions into the Entity-Quality (EQ) formalism, and assigning these EQ descriptions to appropriate taxa (at any rank) [4]. This application motivated us to develop a single taxonomic ontology for vertebrates, the VTO.

The VTO is built as a simple class hierarchy that allows straightforward data aggregation via subsumption reasoning. For example, data referencing the classes ‘Rodentia’ or ‘Primates’ should be returned from a query using ‘Mammalia’. We note, however, that the axioms typically used for linking phenotypic data to taxon classes can have entailments inconsistent with character evolution, for example when phenotypes of a hypothetical common ancestor are evolutionarily lost or reverted to a more primitive state among some of the descendants (see [10] for details). An alternative approach is to view taxonomic entities as historical individuals (e.g., Ghiselin 1974 [11]). Others have explored ontological models that attempt to capture the complex interplay between evolutionary relationships and the practice of taxonomic classification [12,13]. For future work, we intend to make a fuller comparison of the consequences of these two approaches for the kinds of axioms suitable to represent characters that change over evolutionary time.

### Use in the Phenoscape knowledgebase

The VTO was developed to be an integral part of the second-generation Phenoscape Knowledgebase (KB) that is currently under development. Some of the user interface functionality can be seen in prototype form in the first-generation Phenoscape KB (http://kb.phenoscape.org), which uses the TTO and is limited to data from fishes. There is a display page for each taxon that includes its immediate taxonomic parents and children, as well as synonyms, extinction status, and links to other source data for that taxon (e.g., Fishbase, Wikipedia). Taxon display pages also include a browse-able taxonomy tree. Taxa are included in the results returned by queries for phenotypes (e.g., ‘all species with phenotypes in which the basihyal is absent and the pectoral fin is triangular’). Taxa can also be used to scope queries (e.g., ‘all Cyprinidae with triangular fins’) and appear as elements in faceted queries. The Phenoscape KB additionally displays summary phenotype statistics for taxa such as the degree of annotation coverage and phenotypic variation on a simplified taxonomic tree. The representation of the taxonomy as an ontology facilitates aggregation of phenotype annotations at different taxonomic scopes, integrates readily with other ontology-annotated data, and allows reasoning across the entire knowledgebase.

### Maintenance and revision

Harvesting and integration of information from taxonomic sources is driven by curation and research needs. In keeping with the practice recommended for OBO Foundry ontologies [14], curators can submit change requests, for example regarding misspellings or synonyms, through the VTO’s term request tracker (http://purl.obolibrary.org/obo/vto/tracker). If a curator needs to add taxa to or suggest taxonomic rearrangements of the VTO, they are encouraged to contribute this information directly to the source taxonomies following their prescribed curation methodologies. These will then be incorporated back into the VTO when it is updated. Questions and discussions pertaining to the VTO can be directed to the obo-taxonomy mailing list (https://lists.sourceforge.net/lists/listinfo/obo-taxonomy).

### Opportunities for inclusion of additional taxonomic sources

The VTO currently contains 84,179 terminal classes (as of October 2013) and contains the necessary taxa for annotating phenotypes as part of the Phenoscape project. In comparison, the Global Biodiversity Information Facility (GBIF; http://gbif.org) and the Catalogue of Life (http://www.catalogueoflife.org/) contain 84,141 and 65,932 species, respectively, within Chordata. There are additional sources for vertebrate taxonomic information that have not been incorporated in the version of the VTO described here, but would be of value to add in the future. For instance, the Reptile Database (http://www.reptile-database.org/) and the International Ornithologists’ Union Bird List (http://www.worldbirdnames.org) would provide taxa and synonyms not included among the current sources as well as greater resolution to the hierarchy. The IUCN Red List of Threatened Species (http://www.iucnredlist.org/) would provide information about conservation status and potentially widen the application of the ontology. We welcome inquiries from parties interested in integrating these or additional taxonomic resources.

### Comparison to related work

The Global Names project (http://globalnames.org) aggregates taxon checklists and classifications. In contrast to the VTO, it does not create a classification synthesis of its own. The Encyclopedia of Life (EOL) (http://eol.org) also does not synthesize its own classification, but rather indexes its data with each source classification, and then presents the classifications as alternatives. The taxonomy created by GBIF for indexing its aggregated database of species occurrence data is a single taxonomy assembled from over 40 sources in a highly automated way. Its terms of use do not permit free redistribution, and there is no similar mechanism for reporting and addressing quality issues.

For synthesizing a single taxonomy from multiple sources, the Open Tree of Life project (http://opentreeoflife.org) has also developed an almost completely automated approach. It overlays a target taxonomy onto a base taxonomy by retaining all nodes and relationships from the base taxonomy and grafting subtrees from the target taxonomy onto nodes in the base taxonomy where these can be mapped to nodes in the target taxonomy. Due to its large scope, the node mapping is vulnerable to homonymy and other problems, and, in contrast to the Taxonomy Ontology Tool, the synthesis algorithm is not scriptable through a declarative configuration file (J. Rees, pers. comm.).

The tool Phylografter was developed for manipulating phylogenetic trees, including ‘grafting’ one tree onto another [15]. However, it does not support updating as needed for VTO, and it was not designed to handle large-scale taxonomies.

## Conclusions

To fill the need for a single taxonomic ontology including both modern and ancient vertebrate taxa, we have developed the Vertebrate Taxonomy Ontology (VTO) by merging taxonomic information from a variety of expert sources. The integration pipeline we have developed tracks provenance of terms and is capable of incorporating both updates from the source taxonomies and additional sources. Although development to date has been guided by the requirements of the Phenoscape project, we hope that the VTO will be useful for integration of diverse forms of data from vertebrates, and serve as a model for the development of taxonomy ontologies in other groups of organisms.

### Availability and requirements

The Vertebrate Taxonomy Ontology (VTO) and the vocabulary of Taxonomic Ranks that it references are available via their persistent URLs under a Creative Commons Zero (CC0) public domain waiver: VTO, in OWL format, http://purl.obolibrary.org/obo/vto.owl, and in OBO format, http://purl.obolibrary.org/obo/vto.obo; TAXRANK, http://purl.obolibrary.org/obo/taxrank.owl. The VTO is a large ontology, and thus viewing in a desktop OWL editor such as Protégé may require allocating sufficient memory (2 GB at present). The VTO can also be browsed at the NCBO BioPortal (http://bioportal.bioontology.org/ontologies/50317/).

The open-source software used to generate the VTO is available under the MIT license at GitHub (https://github.com/NESCent/Taxonomy-Ontology-Tool), and we welcome further development of this resource by the wider community.

## Competing interests

The authors declare that they have no competing interests.

## Authors’ contributions

Wrote the paper: all authors (PEM, TAD, JPB, WMD, NI, HL, JGL, PMM, PCS, MW, TJV, DCB). Developed and updated ontologies: PEM, JPB, DCB, TAD, NI, WMD, JGL. Developed the figures: TAD, PMM, JPB, PCS. All authors read and approved the final manuscript.

## Authors’ information

Peter E. Midford and T Alex Dececchi are co-first authors.

## References

[B1] Federhen S The NCBI taxonomy database Nucl Acids Res 2013 40 D136 D143 22139910 10.1093/nar/gkr1178 PMC3245000

[B2] Eschmeyer WN Catalog of Fishes California Academy of Sciences http://research.calacademy.org/ichthyology/catalog version 3 2013.

[B3] Mabee PM Balhoff JP Dahdul WM Lapp H Midford PE Vision TJ Westerfield M 500,000 Fish phenotypes: the new informatics landscape of evolutionary and developmental skeletal biology J Appl Ichthy 2012 28 3 300 305 10.1111/j.1439-0426.2012.01985.x PMC3377363 22736877

[B4] Dahdul WM Balhoff JP Engeman J Grande T Hilton E Kothari C Lapp H Lundberg JC Midford PE Vision TJ Westerfield M Mabee PM Evolutionary characters, phenotypes and ontologies: curating data from the systematic biology literature PLoS One 2010 http://dx.doi.org/10.1371/journal.pone.0010708 10.1371/journal.pone.0010708 PMC2873956 20505755

[B5] Fang F Phylogenetic analysis of the Asian cyprinid genus *Danio* (teleostei, cyprinidae) Copeia 2003 2003 714 728 10.1643/IA03-131.1

[B6] Toledo-Piza M The neotropical fish subfamily cynodontinae (teleostei: ostariophysi: characiformes): a phylogenetic study and revision of *Cynodon* and *Rhaphiodon* Amer Mus Nov 2000 3286 1 88

[B7] Froese R Pauly P FishBase World Wide Web electronic publication http://www.fishbase.org, version (04/2013)

[B8] Uhen MD Barnosky AD Bills B Blois J Carrano MT Carrasco MA Erickson GM Eronen JT Fortelius M Graham RW Grimm EC O’Leary MA Mast A Piel WH Polly PD Saila LK From card catalogs to computer: databases in vertebrate paleontology J Vert Paleon 2013 33 1 13 28 10.1080/02724634.2012.716114

[B9] International Commission on Zoological Nomenclature International Code of Zoological Nomenclature International Trust for Zoological Nomenclature 1999 4 London, UK 10.3897/zookeys.931.51583 PMC7205856 32405237

[B10] Balhoff JP Midford PE Lapp H Bodenreider O, Martone ME, Ruttenberg *Integrating Anatomy and Phenotype Ontologies with Taxonomic Hierarchies* Proceedings of the International Conference on Biomedical Ontology 2011 Buffalo, New York, USA 426 427 http://ceur-ws.org/Vol-833

[B11] Ghiselin M A radical solution to the species problem Syst Zool 1974 23 536 544 10.2307/2412471

[B12] Schulz S Stenzhorn H Boeker M The ontology of biological taxa OUP Bioinform 2008 24 i313 i321 10.1093/bioinformatics/btn158 PMC2718636 18586729

[B13] Thau D Franz N Biological taxonomy and ontology development: scope and limitations Biodiv Inform 2010 7 45 66

[B14] Smith B Ashburner M Rosse C Bard J Bug W Ceusters W Goldberg LJ Eilbeck K Ireland A Mungall CJ Consortium OBI Leontis N Rocca-Seraa P Ruttenberg A Sansone SA Scheuermann RH Shah N Whetzel PL Lewis S The OBO Foundry: coordinated evolution of ontologies to support biomedical data integration Nat Biotechnol 2007 25 11 1251 1255 10.1038/nbt1346 17989687 PMC2814061

[B15] Beaulieu JM Ree RH Cavender-Bares J Weiblen GD Donoghue MJ Synthesizing phylogenetic knowledge for ecological research Ecology 2012 93 8s S4 S13

